# ShadowR: a novel chromoprotein with reduced non-specific binding and improved expression in living cells

**DOI:** 10.1038/s41598-019-48604-4

**Published:** 2019-08-19

**Authors:** Hideji Murakoshi, Hiroshi Horiuchi, Takahiro Kosugi, Maki Onda, Aiko Sato, Nobuyasu Koga, Junichi Nabekura

**Affiliations:** 1 Supportive Center for Brain Research, National Institute for Physiological Sciences, Okazaki, Aichi 444-8585 Japan; 2 Department of Physiological Sciences, The Graduate University for Advanced Studies, Hayama, Kanagawa 240-0193 Japan; 3 Department of Structural Molecular Science, The Graduate University for Advanced Studies, Hayama, Kanagawa 240-0193 Japan; 4 Division of Homeostatic Development, National Institute for Physiological Sciences, Okazaki, Aichi 444-8585 Japan; 5 Exploratory Research Center on Life and Living Systems (ExCELLS), Okazaki, Aichi 444-8585 Japan; 6 Research Center of Integrative Molecular Systems, Institute for Molecular Science, Okazaki, Aichi 444-8585 Japan

**Keywords:** Biophysical methods, Molecular biophysics

## Abstract

Here we developed an orange light-absorbing chromoprotein named ShadowR as a novel acceptor for performing fluorescence lifetime imaging microscopy-based Förster resonance energy transfer (FLIM-FRET) measurement in living cells. ShadowR was generated by replacing hydrophobic amino acids located at the surface of the chromoprotein Ultramarine with hydrophilic amino acids in order to reduce non-specific interactions with cytosolic proteins. Similar to Ultramarine, ShadowR shows high absorption capacity and no fluorescence. However, it exhibits reduced non-specific binding to cytosolic proteins and is highly expressed in HeLa cells. Using tandem constructs and a LOVTRAP system, we showed that ShadowR can be used as a FRET acceptor in combination with donor mRuby2 or mScarlet in HeLa cells. Thus, ShadowR is a useful, novel FLIM-FRET acceptor.

## Introduction

Fluorescent proteins are widely used to monitor the localization and dynamics of intracellular proteins in living cells. To detect protein-protein interactions and conformational changes in living cells, Förster resonance energy transfer (FRET) measurement is often used in combination with fluorescent proteins^1–5^. FRET can be monitored when a donor fluorescent molecule is excited and an acceptor molecule is in close proximity (<10 nm). The energy of the excited donor molecule is transferred to the acceptor molecule, and fluorescence is emitted from the acceptor molecule rather than from the donor molecule^4,6–8^. One of quantitative methods to measure and image FRET is fluorescence lifetime imaging microscopy (FLIM) where the fluorescence lifetime of the donor, the time spent in an excited state, is measured and used to quantify FRET^8,9^. As an example of FRET, green fluorescent protein (GFP) and red fluorescent protein (RFP) have often been used as energy donor and acceptor molecules, respectively^10–13^. When GFP is excited, it emits green fluorescence. But, if RFP is located within 10 nm of the GFP molecule, the excitation energy is transferred from GFP to RFP, leading to RFP emission^1,3,5,7,10^. Since GFP fluorescence decreases and RFP fluorescence increases under FRET, the fluorescence intensity changes of both the proteins are used for FRET imaging (i.e., ratiometric imaging) and analysis. When FRET occurs between GFP and RFP, the fluorescence lifetime of GFP is shortened^9,11^. This fluorescence lifetime change can be used as a readout of FRET in FLIM-FRET^11^. One of the characteristics of FLIM-FRET is that it measures only the donor fluorescence, but not the acceptor fluorescence^9,11^. This characteristic enables the use of a dim fluorescent protein as an acceptor for FRET^14^. Dim fluorescent proteins/chromoproteins, which have large extinction coefficients but low quantum yield have been developed and used for FLIM-FRET measurement^14–21^. The advantage of non-fluorescent acceptor proteins is that only donor fluorescence exists, and therefore spectral separation between donor and acceptor fluorescence is not required. Thus, FRET in combination with chromoproteins can be monitored in a narrow bandwidth, facilitating multicolor FRET imaging^14,15,18,19,22^.

Although fluorescent proteins are useful tools in monitoring cellular events, they may be toxic to cells due to non-specific binding to other cellular proteins and inhibition of their activity^23–26^. In addition, when florescent proteins are used to monitor the localization of a certain protein, non-specific interactions of the fluorescent protein could disturb proper localization of the labeled protein. Thus, it is crucial to develop fluorescent proteins or chromoproteins that do not non-specifically bind to cytosolic proteins. Since protein-protein interactions are largely driven by a hydrophobic effect^27,28^, we developed a new chromoprotein as a FLIM-FRET acceptor by replacing the hydrophobic amino acids located at the surface of Ultramarine with hydrophilic ones to reduce non-specific binding to intracellular proteins. The resultant chromoprotein, which we named ShadowR, exhibited reduced interaction with cytosolic proteins and enhanced expression. Furthermore, ShadowR was successfully paired with mRuby2^29^ or mScarlet^30^ for FLIM-FRET measurement in living cells.

## Results

**Figure 1:**
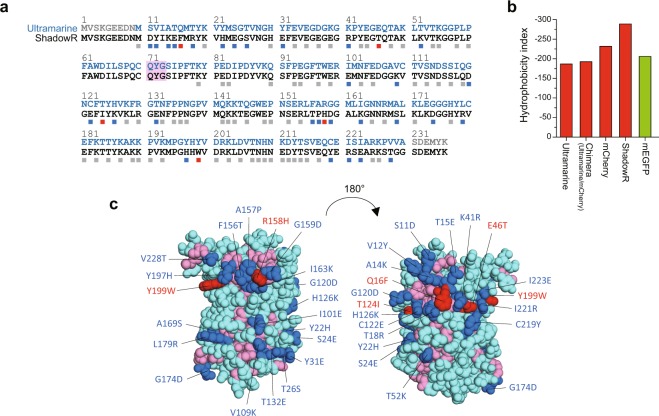
Sequence alignment of Ultramarine and ShadowR. (**a)** Amino acid sequences of Ultramarine and ShadowR are shown in blue and black letters, respectively. Extra amino acids (gray characters) were added to the N and C termini of Ultramarine to match the molecular size of ShadowR. Squares (gray, blue, red) indicate the amino acids (10–228 in Ultramarine/ShadowR) whose side chains are directed outward from the protein. To identify amino acids, we utilized the crystal structure (Protein Data Bank ID: 2H5Q) of mCherry which shares 64% homology to Ultramarine. Blue and Red squares indicate that the amino acids in Ultramarine were replaced to more hydrophilic and hydrophobic amino acids, respectively. Gray squares indicate that the hydrophobicity of the amino acids is identical between Ultramarine and ShadowR. The chromophore tripeptide is highlighted with a magenta box. **(b)** Hydrophobicity index of respective proteins as calculated simply by summing the hydropathy index of amino acids^31^ is indicated by the squares. **(c)** A homology model of ShadowR created by SWISS-MODEL^42^. The X-ray crystal structure of mCherry mutant (PDB ID 3NED)^43^ was used as a template to represent ShadowR. Relatively hydrophobic acids (I, V, L, F, C, M, A, G) are colored in magenta. The rest of amino acids which are the relatively hydrophilic ones (T, S, W, Y, P, N, Q, D, E, H, K, R) are colored in cyan. Blue and Red indicate the amino acids substituted to more hydrophilic or hydrophobic ones in Ultramarine, respectively. For the electrostatic surface potential of Ultramarine and ShadowR, see Supporting Fig. 2S.

To create a novel chromoprotein, we chose Ultramarine as a template^16^. Ultramarine is a monomeric chromoprotein with a relatively large extinction coefficient (64,000 M^−1^cm^−1^) and low quantum yield (~0.001), but has many hydrophobic amino acids on its surface (Fig. 1a). We attempted to make Ultramarine more hydrophilic to reduce its non-specific hydrophobic interaction with cytosolic proteins. To do that, we first created a chimera by replacing the Ultramarine amino acid sequences (1–51, 118–157, and 231–236) with the corresponding regions of mCherry (Fig. S1). We chose this chimera because it is slightly more hydrophilic than Ultramarine (Fig. 1b). The rest of the sequence was not replaced because it surrounds the chromophore and is important for absorption and low quantum yield. Based on the crystal structure data of mCherry (Protein Data Bank ID: 2H5Q), we identified 105 amino acids whose side chains are directed outward from the chimera. Among them, 32 hydrophobic amino acids indicated by green arrowheads in Fig. S1 were selected to be replaced by more hydrophilic ones using single-amino acid saturation mutagenesis, or we replaced to either corresponding mCherry or Ultramarine amino acid (Fig. S1). When the outward directed amino acids are clustered, those are simultaneously subjected to saturation mutagenesis. The PCR products with saturated mutations were ligated into a bacterial expression vector and a genetic library was constructed. To screen the library for hydrophilic chromoproteins, we first identified vivid purple colonies under ambient light, confirming that the mutants have high absorption. We also confirmed that the colonies identified are not fluorescent under blue light illumination. Subsequently, we sequenced the identified colonies and picked mutants with more hydrophilic amino acids than the original sequence. When single-amino acid mutagenesis failed to produce purple colonies, the surrounding amino acids were simultaneously subjected to saturation mutagenesis or replaced to the corresponding Ultramarine or mCherry amino acids. We sequentially repeated this process for the 32 positions (Fig. S1), and finally identified a mutant that shows high absorption and non-fluorescence comparable to Ultramarine (Table 1), but with greater hydrophilicity calculated by using the reported hydropathy index of amino acids^31^ (Fig. 1). As a result of these processes, 19 and 3 amino acids among 32 positions in the Ultramarine/mCherry chimera were replaced to more hydrophilic and hydrophobic ones, respectively (Figs 1c, S1). The three amino acids were replaced to hydrophobic ones, because the replacement of these amino acids to hydrophilic ones resulted in the loss of absorption. Eight amino acids were unchangeable because of the loss of absorption, and 2 amino acids were replaced to the different amino acids, but with the same hydropathy index^31^. The value of surface hydrophobicity of ShadowR is greatly reduced compared to that of Ultramarine or the chimera (Fig. 1b). Furthermore, electrostatic surface potential maps revealed that the electrostatic charge is increased at the surface of ShadowR compared with that Ultramarine (Fig. S2). We named this hydrophilic mutant ShadowR, where R stands for “red”, since the absorption peak (585 nm) is similar to that of red fluorescent proteins.

**Table 1:** Characteristics of ShadowR.

Protein	EC (M^−1^cm^−1^)	QE	Abs (nm)	Ex (nm)	Em (nm)
Ultramarine	64,000^*^	0.001^*^	586^*^	—	626^*^
Ultramarine (this study)	97,700	ND	586	—	—
mCherry_I202Y_	32,000^†^	0.02^†^	590^†^	592^†^	620^†^
ShadowR (this study)	97,100	ND	585	—	—

EC: extinction coefficient, QE: quantum efficiency, Abs: absorption maximum, Ex: excitation maximum, E_m_: emission maximum, ND: not determined. ^*, †^Values obtained from previously published data^16,19^, respectively. Extinction coefficients were measured by the alkaline denaturation method (See Materials and Methods). Since EC and QE measurements could be operation sensitive, we carried out the side-by-side measurement of Ultramarine as a control. The differences in EC values of Ultramarine may be due to differences in experimental conditions or operational differences.

**Figure 2:**
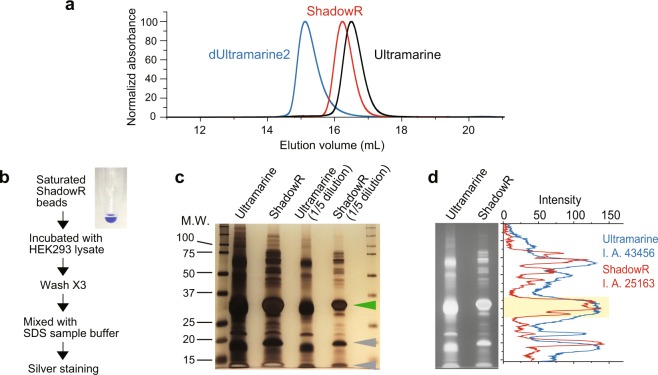
Oligomeric property and non-specific binding of ShadowR to intracellular proteins. **(a**) Size-exclusion chromatography traces of dUltramarine2 (blue), Ultramarine (black), and ShadowR (red). Oligomeric status of ShadowR and Ultramarine was compared. To identify dimer position, dUltramarine2^20^ was used. Molecular weight of dUltramarine2, Ultramarine, and ShadowR are 30913, 30913, and 31365, respectively. Slight difference of the peak positions between ShadowR and Ultramarine may be due to the difference of molecular weight and surface amino acids. **(b)** Schematic drawing of non-specific binding assay. To pulldown the non-specifically bound proteins with Ultramarine or ShadowR, saturated Ultramarine or ShadowR beads are mixed with cell lysate and washed three times (See Materials and Methods). **(c)** Non-specifically bound proteins were separated by SDS-polyacrylamide gel and identified by silver staining. Ultramarine-beads and ShadowR-beads (lane 1, 2) and their diluted sample were also loaded, respectively (lane 3, 4). The green and gray arrowheads indicate His-tagged chromoproteins and their cleaved products, respectively. **(d)** Intensity profiles of band patterns were analyzed. Since the signals of lane 1 and 2 were saturated, lane3 and 4 in c were inverted, and line profiles (background subtracted) were measured and plotted. Integrated area (I. A.) of profiles are also indicated. Since the region indicated by yellow rectangle is the signal of chromoproteins, the regions were not counted for I.A.

Using size-exclusion chromatography, we first confirmed that ShadowR is monomeric similar to Ultramarine (Fig. 2a). To detect the non-specifically bound cytosolic proteins of HEK293 cells to Ultramarine/ShadowR, we developed a new method called the non-specific binding assay (NSB assay). Ni^+^-nitrilotriacetate beads saturated with His-tagged Ultramarine or ShadowR were incubated with cell lysate (Fig. 2b), and non-specifically bound proteins were pulled down by centrifugation. Subsequent silver staining indicated lower levels of non-specific binding of ShadowR to cytosolic proteins than that of Ultramarine (Fig. 2c,d).

**Figure 3:**
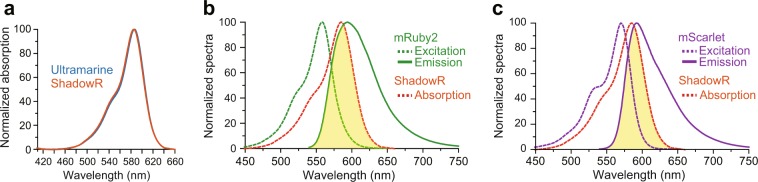
Spectral properties of Ultramarine and ShadowR. (**a)** Normalized absorption spectra of Ultramarine and ShadowR. (**b**,**c**) The spectral overlap (yellow region) between ShadowR absorption spectrum and mRuby2 **(b)** and mScarlet **(c)** emission spectra.

Spectral analysis of purified ShadowR confirmed that, similar to Ultramarine, ShadowR has an excitation peak at 585 nm, and molar extinction coefficient of 97,100 M^−1^·cm^−1^ (Fig. 3a and Table 1). Quantum efficiency was not measurable because of the lack of fluorescence. Since the absorption spectrum of ShadowR significantly overlaps with the emission spectrum of mRuby2 (Fig. 3b) and mScarlet (Fig. 3c), mRuby2/ShadowR and mScarlet/ShadowR may serve as FRET pairs.

**Figure 4:**
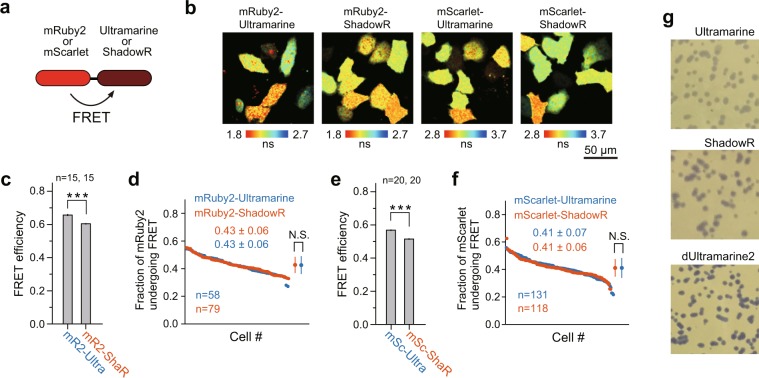
FRET efficiency and maturity of ShadowR in tandem fluorescent proteins. **(a)** A schematic drawing of the constructs used to evaluate the FRET efficiency and fraction of the mRuby2 or mScarlet undergoing FRET. **(b)** Representative fluorescence lifetime images of the tandem proteins in HeLa cells; the images were taken at 1000-nm two-photon excitation. Because the expression level of mRuby2-Ultramarine and mScarlet-Ultramarine was low, we used different laser powers for each condition (5 mW for mRuby2-Ultramarine, 4 mW for mRuby2-ShadowR, 3 mW for mScarlet-Ultramarine, 2 mW for mScarlet-ShadowR). Scale bar, 50 µm. **(c**,**e)** Quantification of FRET efficiency of the tandem proteins. The fluorescence lifetime over the whole image was used for the analysis (See Materials and Methods). The number of images used for the analysis are indicated in the figure. Each image contains 4–12 cells, and the data are presented as mean ± SEM. Asterisks denote statistical significance (*t* test, *P < 0.05, **P < 0.01, ***P < 0.001, N.S.  = not significant). **(d**,**f)** A comparison of the fraction of mRuby2 or mScarlet fluorescent protein undergoing FRET (chromophore maturation efficiency of Ultramarine or ShadowR) analyzed in individual cells; data were plotted in the descending order. The FRET fraction is directly related to the maturation efficiency of an acceptor, i.e., Ultramarine **(d)** or ShadowR **(f)**. Means ± SD are also plotted on the right (*t* test, *P < 0.05, **P < 0.01, ***P < 0.001, N.S.  = not significant). The number of samples (n) and mean ± SD are also indicated. (**g**) Comparison of E. coli expressing Ultramarine, ShadowR, or dUltramarine2. Purple colonies indicate that the *E. coli* cells express the respective chromoproteins. After transformation, the cells were incubated for 16 hr at 32 °C and imaged.

To quantify the performance of mRuby2/ShadowR and mScarlet/ShadowR pairs as FRET pairs, we measured FRET efficiency and maturation efficiency using fusion proteins in HeLa cells and compared the results with those of ShadowR and Ultramarine proteins, as described previously (Fig. 4a)^18^. We expressed tandem constructs by lipofection, and measured the fluorescence lifetime of mRuby2 or mScarlet in the living HeLa cells by 2-photon FLIM-FRET (Fig. 4a–f). We used 2-photon excitation for imaging because of its low phototoxicity compared with single-photon excitation^32^. Because of the low levels of expression of Ultramarine fusions (i.e., mRuby2/Ultramarine and mScarlet/Ultramarine) compared with those of ShadowR, we used a higher laser power for their imaging (See Fig. 4b legend). By analyzing the fluorescence lifetime decay curves, we measured the FRET efficiency and maturity of each acceptor separately, as described earlier^9,11,15^. While the FRET efficiencies of mRuby2 and mScarlet fusions with ShadowR were lower than those with Ultramarine (Fig. 4c,e), the maturity of ShadowR fusion proteins was comparable to that of Ultramarine fusion proteins (Fig. 4d,f). The slight decrease in FRET efficiency with ShadowR could be due to the difference in the relative orientation of the chromophore with regard to the donor and the acceptor, because of the amino acid sequence difference between Ultramarine and ShadowR. Next, we observed the chromophore maturation of ShadowR in *E. coli* (Fig. 4g) and found that the colonies expressing ShadowR exhibit more vivid purple color than those expressing Ultramarine, suggesting that ShadowR has better maturation in *E. coli*.

**Figure 5:**
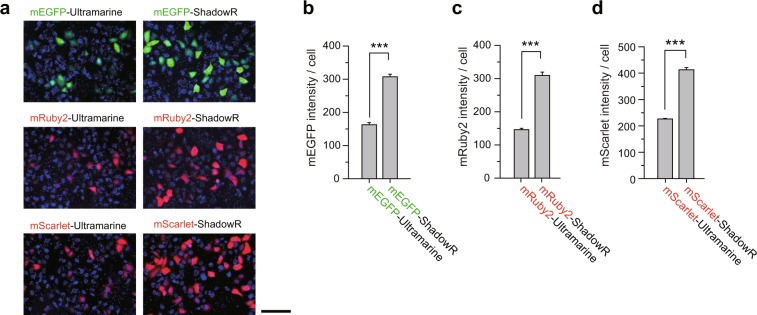
Epifluorescence analysis of Ultramarine and ShadowR fusion proteins. **(a)** Tandem constructs of mEGFP (green), mRuby2 (red), and mScarlet (red) with Ultramarine or ShadowR were expressed in HeLa cells and imaged under an epifluorescence microscope. All cells in the image field were identified by Hoechst 33342 staining (blue). Scale bar, 100 µm. **(b**–**d)** The fluorescence intensities of mEGFP **(b)**, mRuby2 **(c)**, and mScarlet **(d)** in the image field were measured and divided by the cell number determined by Hoechst staining and counting. Ten images per condition were used for the analysis. Each image contains 500–1000 cells, and the data are presented as mean ± SEM. Asterisks denote statistical significance (*t* test, *P < 0.05, **P < 0.01, ***P < 0.001, N.S.  = not significant).

Next, we confirmed the expression of ShadowR in HeLa cells. Since ShadowR has no fluorescence, we fused A206K-mutated monomeric EGFP (mEGFP)^33^, mRuby2, or mScarlet with ShadowR to visualize ShadowR expression as fusion protein. The fluorescence level from individual cells was quantified by epifluorescence microscopy (Fig. 5). The cells expressing mEGFP/ShadowR showed higher fluorescence intensities compared with those expressing mEGFP/Ultramarine (Fig. 5a,b). Similar results were obtained with mRuby2 and mScarlet fusion proteins (Fig. 5a,c,d). Since there is spectral overlap between mScarlet/mRuby2 emission and ShadowR absorption (Fig. 3b,c), the increased brightness of mScarlet/mRuby2 could be due to the lower levels of complete maturation of ShadowR compared with Ultramarine. However, this possibility is excluded since the maturation and FRET efficiency of ShadowR is comparable to those of Ultramarine (Fig. 4c–f).

**Figure 6:**
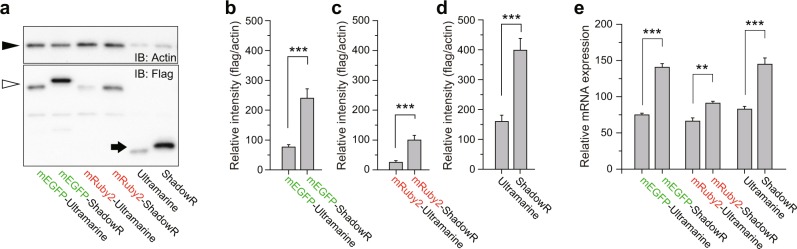
Protein and mRNA expression assay of Ultramarine and ShadowR fusion constructs. **(a)** HeLa cells expressing tandem constructs of mEGFP, mRuby2, and mScarlet with Ultramarine or ShadowR were lysed and immunoblotted with anti-FLAG and anti-β-actin antibodies. **(b**–**d)** Quantitative analysis of immunoblotting. The band intensities of FLAG-tagged fluorescent and chromo proteins were divided by those of actin. Error bars indicate S.E.M. for seven independent experiments. Asterisks denote statistical significance (*t* test, *P < 0.05, **P < 0.01, ***P < 0.001, N.S.  = not significant). **(e)** The expression of indicated genes in HeLa cells was quantified with qRT-PCR. As an endogenous reference gene, beta-actin mRNA levels were measured. Asterisks denote statistical significance (*t* test, *P < 0.05, **P < 0.01, ***P < 0.001, N.S.  = not significant).

Since the tandem constructs with ShadowR exhibited brighter fluorescence (Fig. 5), we next performed western blotting and real time PCR to determine if the increased fluorescence observed with ShadowR was due to increased protein or mRNA expression (Fig. 6). Western blotting revealed increased expression of mEGFP/ShadowR and mRuby2/ShadowR fusion proteins (Fig. 6a–c). Non-fused ShadowR also showed increased expression compared to Ultramarine (Fig. 6a,d). Slight band shift compared with Ultramarine fusion was observed for mEGFP/ShadowR and ShadowR. We rigorously checked their DNA sequences and confirmed that there is no unwanted insertion. Most likely, the band shift was due to the replacement of hydrophobic amino acids with charged hydrophilic amino acids or increased molecular weight (See Fig. 2a legend). Next, we quantified the mRNA levels of each construct, and found higher mRNA expression levels of ShadowR, mEGFP/ShadowR, and mRuby2/ShadowR than those of Ultramarine and its fusions (Fig. 6e). These results suggested that ShadowR exhibits enhanced mRNA and protein expression.

**Figure 7:**
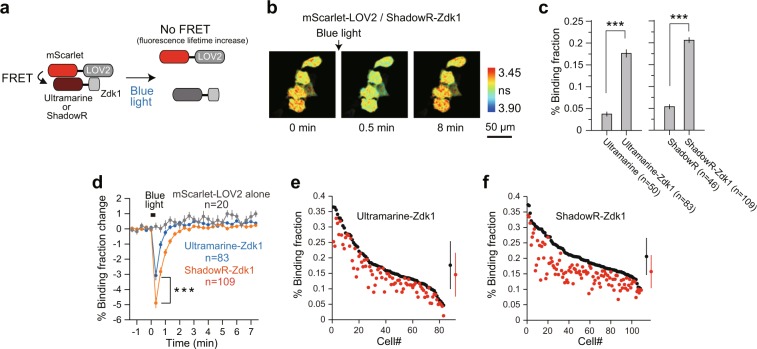
The performance of ShadowR in a LOVTRAP system in HeLa cells. **(a**) A schematic of the LOVTRAP FRET construct. **(b**) Representative fluorescence lifetime images of mScarlet-LOV2 paired with ShadowR-Zdk1 in HeLa cells after blue light illumination (2 s at 35 mW/cm^2^). Scale bar, 50 µm. **(c)** A comparison of the fraction of mScarlet-LOV2 undergoing FRET paired with chromoproteins (Ultramarine/ShadowR) alone or with Zdk1 in the absence of blue light. The number of cells (n) analyzed are indicated. The data are presented as mean ± SEM (*t* test, *P < 0.05, **P < 0.01, ***P < 0.001, N.S.  = not significant). **(d)** An averaged time course of binding fraction changes in response to blue light (blue; mScarlet-LOV2/Ultramarine-Zdk1, orange; mScarlet-LOV2/ShadowR-Zdk1, gray; mScarlet-LOV2 alone). The number of cells (n) analyzed are indicated. The data are presented as mean ± SEM (*t* test, *P < 0.05, **P < 0.01, ***P < 0.001, N.S. = not significant). **(e**,**f)** The lifetime changes in individual HeLa cells before and after light illumination (the same dataset as in panels c). The basal fluorescence lifetime (averaged over −1.3 to 0 min) of individual cells is plotted in the descending order (black) along with the corresponding fluorescence lifetime values (at 20 sec) after blue-light illumination (red). The data are also presented as mean ± SD on the right.

We further tested the performance of ShadowR using a genetically encoded optogenetic tool, the LOVTRAP system^34^. The LOVTRAP system consists of Zdk1 and LOV2 domains^35,36^, and their dissociation and association can be controlled by blue light. We fused mScarlet with LOV2 and ShadowR or Ultramarine with Zdk1, creating a LOVTRAP FRET construct (Fig. 7a). These pairs were expressed in HeLa cells and their blue light-dependent association and dissociation were imaged and quantified by 2pFLIM-FRET (Fig. 7b). We only tested mScarlet-LOV2, not mRuby2-LOV2, because mScarlet is much brighter in cells than mRuby2. In the absence of blue light, mScarlet-LOV2 bound to Ultramarine/ShadowR-Zdk1 in cells, but not Ultramarine/ShadowR alone, suggesting that Zdk1 binds to mScarlet-LOV2 (Fig. 7c). Next, HeLa cells expressing the LOVTRAP FRET construct were illuminated with blue light at 35 mW/cm^2^ for 2 s. Immediately after illumination, the fluorescence lifetime of mScarlet in LOV2 increased by decreased FRET, and returned to basal levels in approximately 60 s, consistent with results of another study^34^. The binding fraction change (i.e., the fraction of mScarlet bound to Zdk1, see also Materials and Methods) of mScarlet-LOV2/ShadowR-Zdk1 was larger than that of mScarlet-LOV2/Ultramarine-Zdk1, suggesting that ShadowR is a superior chromoprotein in the LOVTRAP system. As a control experiment, we only expressed mScarlet-LOV2 and found that there was no binding fraction change after light illumination, suggesting that the change in binding fraction was due to the dissociation of Zdk1 (Fig. 7d–f).

## Discussion

Here, we successfully developed a new chromoprotein, ShadowR, as a FLIM-FRET acceptor for pairing with mScarlet or mRuby2. Compared with the previously reported chromoprotein, Ultramarine^16^, ShadowR has superior property in terms of reduced non-specific binding to cellular proteins (Fig. 2b–d). The observed reduced non-specific binding of ShadowR is most likely due to the increased hydrophilic property compared with Ultramarine. Another feature of ShadowR is its increased protein expression in HeLa cells (Figs 5, 6), that facilitates imaging with lower laser power for reduced photodamage. It is not currently known if there is the causal association between the increased expression and the surface hydrophilicity (Fig. 6a–d). Since the protein expression tend to correlate with mRNA expression^37,38^, the increased protein may be due to the increased levels of mRNA (Fig. 6e). A few possibilities should be considered regarding the mechanism underlying increased mRNA expression: 1) the expressed Ultramarine proteins inhibit the transcription machinery of mRNA (negative feedback) or 2) the transfected Ultramarine DNA adopts a structure that leads to inefficient transcription. However, the precise mechanism underlying increased mRNA levels is unknown and difficult to deduce from our experiments.

We previously reported the use of dark mCherry, mCherry_I202Y_, as a FRET acceptor^19^. The advantage of ShadowR over dark mCherry is its darkness. While the quantum efficiency of the dark mCherry is 0.02, the quantum efficiency of ShadowR is undetectable. This superior darkness may prevent artificial FRET signals due to fluorescence contamination^18^. The application of ShadowR to a LOVTRAP system yielded a large FRET changes, compared with those of the Ultramarine version of constructs (Fig. 7). The reason of the enhanced FRET change could be due to reduced non-specific interactions of ShadowR with mScarlet or LOV2 compared with Ultramarine. In the dark state, mScarlet-LOV2 binds to ShadowR-Zdk1 via LOV2 and Zdk1. However, if additional non-specific interactions such as the binding of ShadowR to mScarlet or LOV2 exists and are weaker than those of Ultramarine due to the surface amino acid difference, the light-dependent separation of ShadowR-Zdk1 from mScarlet-LOV2 compared with that of Ultramarine fusions is facilitated. While ShadowR constructs exhibit the significant FRET signals, they show quite large cell-to-cell variability (Figs 4d, f, 7e, f). One of the future directions for improving ShadowR is to minimize this variability for more accurate measurement.

Taken together, we believe that ShadowR will be an additional useful tool for these studies, especially for FLIM-FRET.

## Materials and Methods

### Saturation mutagenesis

The synthesized gene encoding Ultramarine was purchased from FASMAC (Kanagawa, Japan). This gene in a customized pRSET vector (Invitrogen) was used as a template for constructing genetic libraries for ShadowR development. Sequential saturation mutagenesis to the targeted positions was performed by PCR amplification with degenerate primers in combination with overlapping PCR. Subsequently, the amplicons were subcloned into the customized pRSET vector. For making a library, the plasmid library was introduced into electro- or chemically competent cells, and the cells were grown for 18–20 h at 34 °C on LB agar plates supplemented with antibiotics.

### Plasmid construction for mammalian expression

For all DNA construction described below, a modified pEGFP-C1 plasmid (Clontech), where a kanamycin resistance gene was replaced with an ampicillin resistance gene, was used as a backbone vector. The synthesized *LOV2*, *Zdk1*, and *mScarlet* genes were purchased from FASMAC (Kanagawa, Japan). The *mRuby2* gene construct was a gift from Michael Lin (Addgene plasmid #40255). For *Ultramarine* and *ShadowR*, the respective genes were inserted into the vector by replacing EGFP. Extra sequences encoding amino acid sequences MVSKGEEDN and SDEMYK were fused to the N and C termini of *Ultramarine*, respectively, so it would match the molecular weight of ShadowR for reasonable comparison during experiments (Fig. 1a). To construct tandem protein plasmids, the *Ultramarine* (DNA sequence encoding amino acid residues 1–214) or *ShadowR* (1–214) gene was ligated with FLAG-tagged *mEGFP* (1–232, A206K-mutated monomeric EGFP), *mRuby2* (1–229), or *mScarlet* plasmid (1–224) with a linker encoding the peptide VDGTAGPGSG. These tandem plasmids were used for experiments shown in Figs 4–6.

To construct LOVTRAP system-based FRET constructs, we fused FLAG-tagged *mScarlet2* (DNA sequence corresponding to amino acid residues 1–232) with the N terminus of the LOV2 domain (DNA sequence corresponding to amino acid residues 404–546 in phototropin) with a linker encoding the peptide SGLRS and used this as a donor for FRET. As an acceptor, FLAG-tagged *Ultramarine* (1–214) or *ShadowR* (1–214) genes were fused to the N terminus of Zdk1 with no linker.

### Spectral properties of the chromoproteins

His-tagged chromoproteins were overexpressed in *Escherichia coli* DH5α cells and purified on an Ni^+^-nitrilotriacetate column (HiTrap, GE Healthcare). Mature protein concentrations were calculated from the extinction coefficient of the chromophore after denaturation in 0.1 N NaOH (44,000 M^−1^·cm^−1^ at 452 nm)^39^. Absorption spectra of the proteins diluted in PBS were recorded on a spectrophotometer (UV-1800; Shimadzu). The extinction coefficients of fluorescent proteins were determined by dividing the peak optical density by the molar concentration of matured proteins.

### Cell culture and transfection

HeLa cells were cultured in Dulbecco’s modified Eagle’s medium (DMEM) supplemented with 5% fetal bovine serum (FBS) at 37 °C and 5% CO_2_. The cells were transfected with the plasmids using Lipofectamine 3000 (Invitrogen), followed by incubation for 16–20 hr in the absence of serum. Two-photon FLIM-FRET imaging was conducted in a solution containing 4-(2-hydroxyethyl)-1-piperazineethanesulfonic acid (HEPES; 30 mM, pH 7.3)-buffered artificial cerebrospinal fluid (130 mM NaCl, 2.5 mM KCl, 1 mM CaCl_2_, 1 mM MgCl_2_, 1.25 mM NaH_2_PO_4_, 25 mM glucose) at room temperature (23–35 °C).

### Non-specific binding assay

A saturable amount of purified Ultramarine or ShadowR was bound to Ni^+^-nitrilotriacetate beads (HiTrap, GE Healthcare), respectively, and the beads were washed three times with phosphate buffered saline (PBS) to wash out free proteins. To prepare HEK293 lysate, cells cultured in a 15 cm dish were trypsinized and suspended in DMEM supplemented with 5% FBS. Subsequently, cells were precipitated by centrifugation and suspended in 10 ml of PBS. The suspended cells were disrupted by sonicating on ice for 10 s at 30 W using an ultrasonic disruptor (UD-211, TOMY) and centrifuged. Then, supernatant was filtered with a 0.22 μm filter membrane. For the non-specific binding assay, 20 μl of ShadowR-bead slurry was mixed and incubated with 500 μl of HEK293 lysate for 20 min at room temperature. The beads were washed three times with PBS. A brief sonication was performed during each wash. Samples were dissolved in SDS sample buffer. Then, silver staining was performed using a silver stain reagent kit (Cosmo Bio).

### Size-exclusion chromatography

Size-exclusion chromatography was carried out using a Superdex 200 Increase 10/300 GL column (GE Healthcare) on an AKTA pure 25 chromatography system (GE Healthcare). Proteins purified Ni-NTA column (500 μl with concentration of 40 μM) were subjected to gel filtration chromatography at the flow rate of 0.5 ml/min with PBS. To detect chromoproteins, 280 nm was used.

### RT-PCR

Total RNAs from HeLa cells were isolated with an RNeasy Mini Kit (Qiagen, Valencia, CA, USA), and were reverse-transcribed with the Transcriptor First Strand cDNA Synthesis Kit (Roche, Indianapolis, IN, USA). Expression levels of mRNAs encoding FLAG were assessed by quantitative PCR using FastStart Essential DNA Green Master (Roche) on Step One real-time PCR system (Life Technologies, Carlsbad, CA, USA). All primers were obtained from FASMAC (Kanagawa, Japan), their sequences were as follows:

Forward for flag: 5′-CGGCCGCGACACTAGATCA-3′;

Reverse for flag: 5′-ATGTTTCAGGTTCAGGGGGAG-3′;

Forward for β-actin: 5′-CGGCGCCCTATAAAACCCA-3′;

Reverse for β-actin: 5′-ATCATCCATGGTGAGCTGGC-3′;

Gene expression values were calculated by the delta-delta Ct method. Assays were carried out in five independent trials.

### Epifluorescence imaging

Hoechst 33342 was purchased from Dojindo. HeLa cells expressing fluorescent proteins were incubated with 1 μg/ml Hoechst for 10 min. Subsequently, the cells were observed under an epifluorescence microscope. For excitation, a blue light (475 nm LED; CoolLED) for mEGFP, a green light (565 nm LED; CoolLED) for mRuby2 and mScarlet, or a purple light (365 nm LED; CoolLED) for Hoechst was used. The fluorescence images were taken with a sCMOS camera (ZYLA 4.2; Andor) mounted on a microscope (BX51WI; Olympus) through a 20x objective lens.

### Two-photon fluorescence lifetime imaging

A custom-made two-photon fluorescence lifetime imaging microscope was used. Briefly, mRuby2 or mScarlet as the FRET construct was excited with a Ti-sapphire laser (Mai Tai; Spectra-Physics) tuned to 1000 nm. The X/Y scan mirrors (6210H; Cambridge Technology) were controlled with ScanImage software^40^. The fluorescence photon signals from cells were collected with an objective lens (60x, 1.0 NA; Olympus) and a photomultiplier tube (H7422-40p; Hamamatsu) placed after a dichroic mirror (FF553-SDi01; Semrock) and emission filter (FF01-625/90; Semrock). For blue light illumination in the LOVTRAP experiment (Fig. 7), blue LED (244-87-470-50E-40; CoolLED) with a band pass filter (FF01-469/35-25; Chroma) was used. A fluorescence lifetime curve was recorded by a time-correlated single-photon-counting board (SPC-150; Becker & Hickl) controlled with custom software^11^. To construct a fluorescence lifetime image, the mean fluorescence lifetime values <*t*> in each pixel were calculated using Eq. (1) and translated into a color-coded image^11^:

1 $$ < t\, > =\int \,tF(t)dt\div\int \,F(t)dt-{t}_{0}$$

where *t*_*o*_ is obtained by fitting the whole image with single exponential or double exponential functions convolved with an instrument response function as described in the following section.

### Quantification of FRET efficiency and maturity

To compare FRET efficiency between mScarlet/mRuby2 and ShadowR/Ultramarine in HeLa cells, we fitted the fluorescence lifetime curve with a double exponential function convolved with an instrument response function, *G*(*t*), assuming that two fractions exist in the cells: (1) mature donor fluorescent protein (i.e., mScarlet or mRuby2) fused to an immature acceptor fluorescent protein (i.e., ShadowR or Ultramarine); (2) mature donor fused to a mature acceptor where FRET occurs and the fluorescence lifetime of the donor gets shorter:

2 $${\rm{F}}({\rm{t}})={{\rm{P}}}_{{\rm{free}}}\exp (\frac{{{\rm{\sigma }}}_{{\rm{G}}}^{{\rm{2}}}}{{{2{\rm{\tau }}}_{{\rm{free}}}}^{{\rm{2}}}}-\frac{{\rm{t}}-{{\rm{t}}}_{{\rm{0}}}}{{{\rm{\tau }}}_{{\rm{D}}}}){\rm{erfc}}(\frac{{{\rm{\sigma }}}_{{\rm{G}}}^{{\rm{2}}}-{{\rm{\tau }}}_{{\rm{free}}}({\rm{t}}-{{\rm{t}}}_{{\rm{0}}})}{\sqrt{{\rm{2}}}{{\rm{\tau }}}_{{\rm{free}}}{{\rm{\sigma }}}_{{\rm{G}}}})+{{\rm{P}}}_{{\rm{FRET}}}\exp (\frac{{{\rm{\sigma }}}_{{\rm{G}}}^{{\rm{2}}}}{{{2{\rm{\tau }}}_{{\rm{FRET}}}}^{{\rm{2}}}}-\frac{{\rm{t}}-{{\rm{t}}}_{{\rm{0}}}}{{{\rm{\tau }}}_{{\rm{FRET}}}}){\rm{erfc}}(\frac{{{\rm{\sigma }}}_{{\rm{G}}}^{{\rm{2}}}-{{\rm{\tau }}}_{{\rm{FRET}}}({\rm{t}}-{{\rm{t}}}_{{\rm{0}}})}{\sqrt{{\rm{2}}}{{\rm{\tau }}}_{{\rm{FRET}}}{{\rm{\sigma }}}_{{\rm{G}}}})$$

In Eq. (2), erfc is a complementary error function, t_0_ is the time offset, σ_G_ is the standard deviation of the IRF, and P_free_ and P_FRET_ are the populations of the free donor (i.e., donor fused to immature acceptor) and donor with FRET (i.e., donor fused to matured acceptor), respectively. τ_free_ and τ_FRET_ are the fluorescence lifetime of free donor and donor with FRET, respectively^9,11,41^. τ_free_ can be independently measured (mScarlet (3.69 ns), mRuby2 (2.45 ns)). By fixing these values in Eq. (2), we obtained τ_FRET_ values for mRuby2-Ultramarine (0.84 ns), mRuby2-ShadowR (0.97 ns), mScarlet-Ultramarine (1.60 ns), and mScarlet-ShadowR (1.79 ns). The mean FRET efficiency (*Y*_FRET_) between the donor and the mature acceptor was calculated as follows:

3 $${Y}_{{\rm{FRET}}}=1-\frac{{{\rm{\tau }}}_{{\rm{FRET}}}}{{{\rm{\tau }}}_{{\rm{free}}}}$$

Using the obtained τ_free_ and τ_FRET_ values, we calculated the fraction of the mature acceptor or the binding fraction (donor undergoing FRET) in individual cells using the following formula as described elsewhere^9,11^:

4 $${{\rm{P}}}_{{\rm{FRET}}}=\frac{{{\rm{\tau }}}_{{\rm{free}}}({{\rm{\tau }}}_{{\rm{free}}}-{{\rm{\tau }}}_{{\rm{m}}})}{{({\rm{\tau }}}_{{\rm{free}}}-{{\rm{\tau }}}_{{\rm{FRET}}}{)({\rm{\tau }}}_{{\rm{free}}}+{{\rm{\tau }}}_{{\rm{FRET}}}-{{\rm{\tau }}}_{{\rm{m}}})}$$

## Supplementary information

Supplementary information

## Data Availability

The data generated and analyzed during the current study are included in this published article and its supplementary information files. Datasets are available from the corresponding author on request.
