# Supplementary material for: ShadowR: a novel chromoprotein with reduced non-specific binding and improved expression in living cells
Source: Sci Rep. 2019 Aug 19;9:12072. doi: 10.1038/s41598-019-48604-4 (PMC6700193; doi:10.1038/s41598-019-48604-4)
Supplement: Supplementary file 1 — Supplementary information [file 41598_2019_48604_MOESM1_ESM.pdf]

Supplementary information

ShadowR: a novel chromoprotein with reduced non-specific binding and improved expression in living cells

Hideji Murakoshi[1,2,*], Hiroshi Horiuchi[2,4,†], Takahiro Kosugi[3,5,6,†], Maki Onda[1], Aiko Sato[1], Nobuyasu Koga[3,5,6], Junichi Nabekura[2,4]

[1] Supportive Center for Brain Research, National Institute for Physiological Sciences, Okazaki, Aichi 444-8585, Japan, [2] Department of Physiological Sciences/[3]Structural Molecular Science, The Graduate University for Advanced Studies, Hayama, Kanagawa 240-0193, Japan, [4] Division of Homeostatic Development, National Institute for Physiological Sciences, Okazaki, Aichi 444-8585, Japan, [5] Exploratory Research Center on Life and Living Systems (ExCELLS), Okazaki, Aichi 444-8585, Japan, [6] Research Center of Integrative Molecular Systems, Institute for Molecular Science, Okazaki, Aichi 444-8585, Japan

† These authors equally contributed to this work

*To whom correspondence should be addressed: Hideji Murakoshi, Supportive Center for Brain Research, National Institute for Physiological Sciences, Okazaki, Aichi 444-8585, Japan; Tel.: +81 564-55-7857; Fax: +81 564-55-7858; e-mail: murakosh@nips.ac.jp

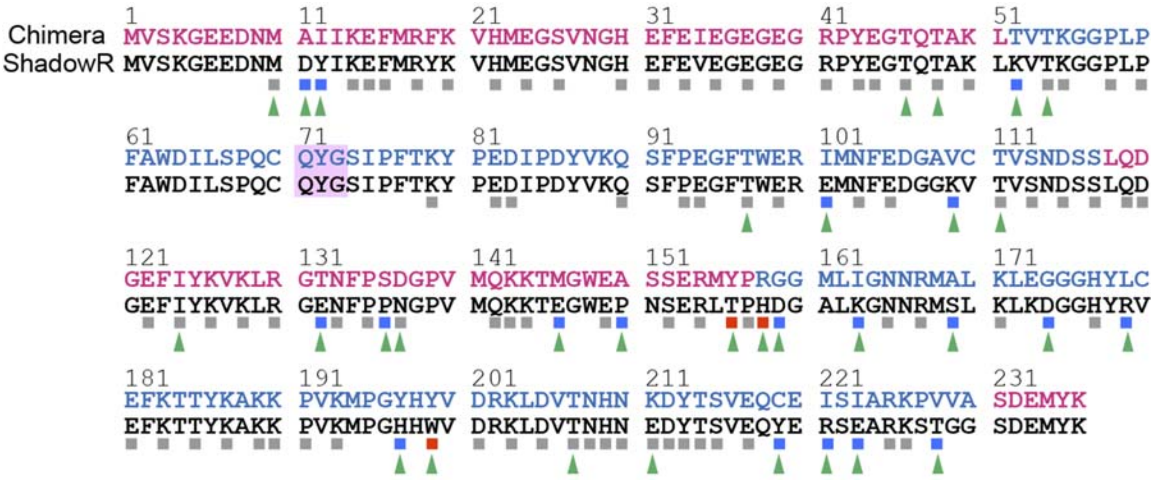

Figure S1. Sequence alignment of Ultramarine/mCherry chimera and ShadowR.

Amino acid sequences of a chimera (Ultramarine sequences, blue letters; mCherry sequences, magenta letters) and ShadowR (black letters) are shown. Squares (gray, blue, red) indicate the amino acids (10–228) whose side chains are directed outward from the proteins. Green arrowheads (32 positions) indicate the positions where saturation mutagenesis or the replacement to either mCherry or Ultramarine amino acid were carried out. When single-amino acid mutation failed to produce purple colonies, the surrounding amino acids were simultaneously subjected to saturation mutagenesis. In addition, F19Y/M161A and I34V mutations were introduced during early and late stage of the ShadowR development, respectively, because *E. coli* colonies expressing the mutants with these mutations exhibited more dense purple color. If an amino acid indicated by a green arrowhead is identical between chimera and ShadowR, it means the amino acid was unchangeable due to the loss of light absorption. Blue (19 positions) and Red (3 positions) squares indicate that the amino acids in the chimera were replaced to more hydrophilic and hydrophobic amino acids in ShadowR, respectively. Gray squares indicate that the hydrophobicity of the amino acids is identical between the chimera and ShadowR. The chromophore tripeptide is highlighted with a magenta box.

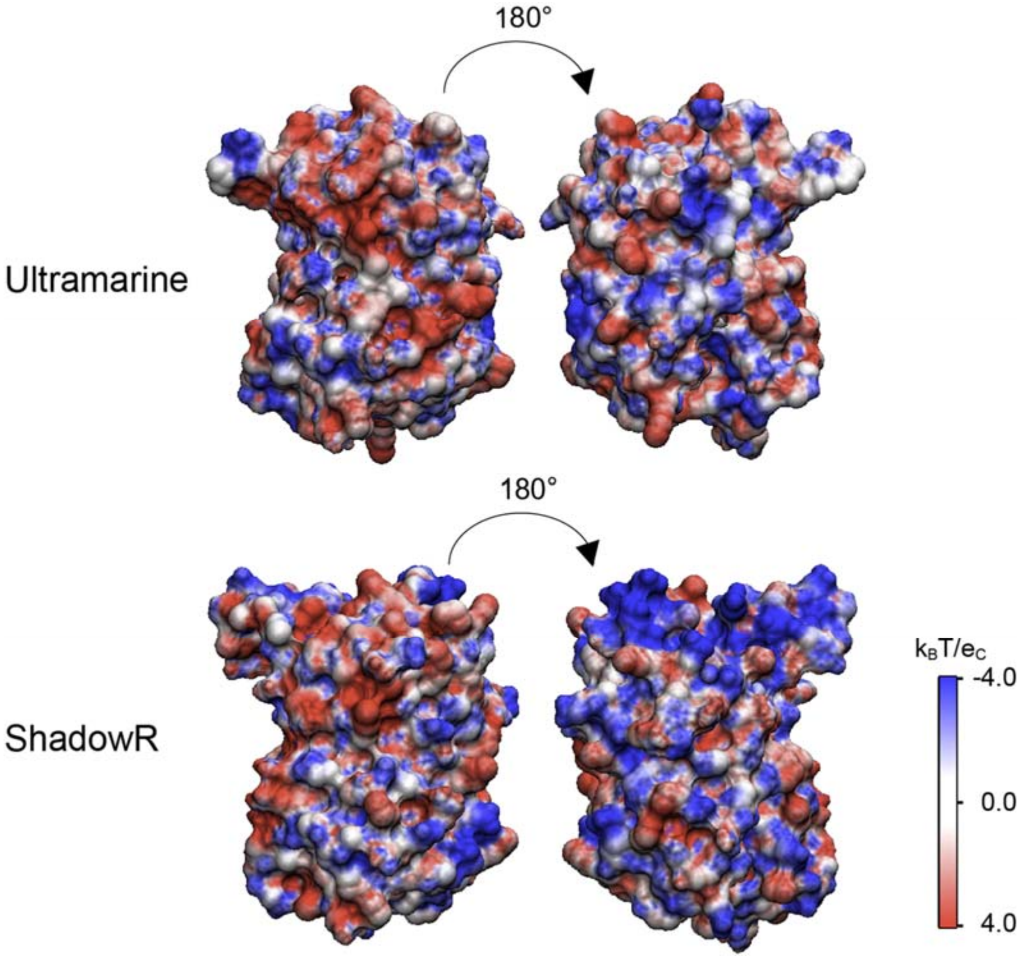

Figure S2. Electrostatic surface views of Ultramarine and ShadowR.

Electrostatic surface potentials were calculated using APBS (Adaptive Poisson-Boltzmann Solver) plugin in the VMD[1] with the nonlinear Poisson-Boltzmann equation and contoured at ± 4 k_BT/e_C. The homology models of Ultramarine and ShadowR based on the crystal structure of mCherry mutant (PDB ID 3NED)[2] and Rtms5 mutant (PDB ID 2ARL)[3] were used, respectively. Negatively and positively charged surface areas are colored blue and red, respectively. Note that the surface area colored with blue is increased in ShadowR compared with that of Ultramarine.

1 Humphrey, W., Dalke, A. & Schulten, K. VMD: visual molecular dynamics. *J Mol Graph* **14**, 33-38, 27-38 (1996).
2 Chica, R. A., Moore, M. M., Allen, B. D. & Mayo, S. L. Generation of longer emission wavelength red fluorescent proteins using computationally designed libraries. *Proc Natl Acad Sci U S A* **107**, 20257-20262, doi:10.1073/pnas.1013910107 (2010).
3 Wilmann, P. G. *et al.* The 2.0 angstroms crystal structure of a pocilloporin at pH 3.5: the structural basis for the linkage between color transition and halide binding. *Photochem Photobiol* **82**, 359-366, doi:10.1562/2005-05-02-RA-509 (2006).
